# Supplementary material for: Mutant p53 Depletion by Novel Inhibitors for HSP40/J-Domain Proteins Derived from the Natural Compound Plumbagin
Source: Cancers (Basel). 2022 Aug 29;14(17):4187. doi: 10.3390/cancers14174187 (PMC9454493; doi:10.3390/cancers14174187)
Supplement: Supplementary file 1 [file cancers-14-04187-s001.zip › cancers-1822168-Supplementary Figure S7.pdf]

A Original blots of Figure 1A

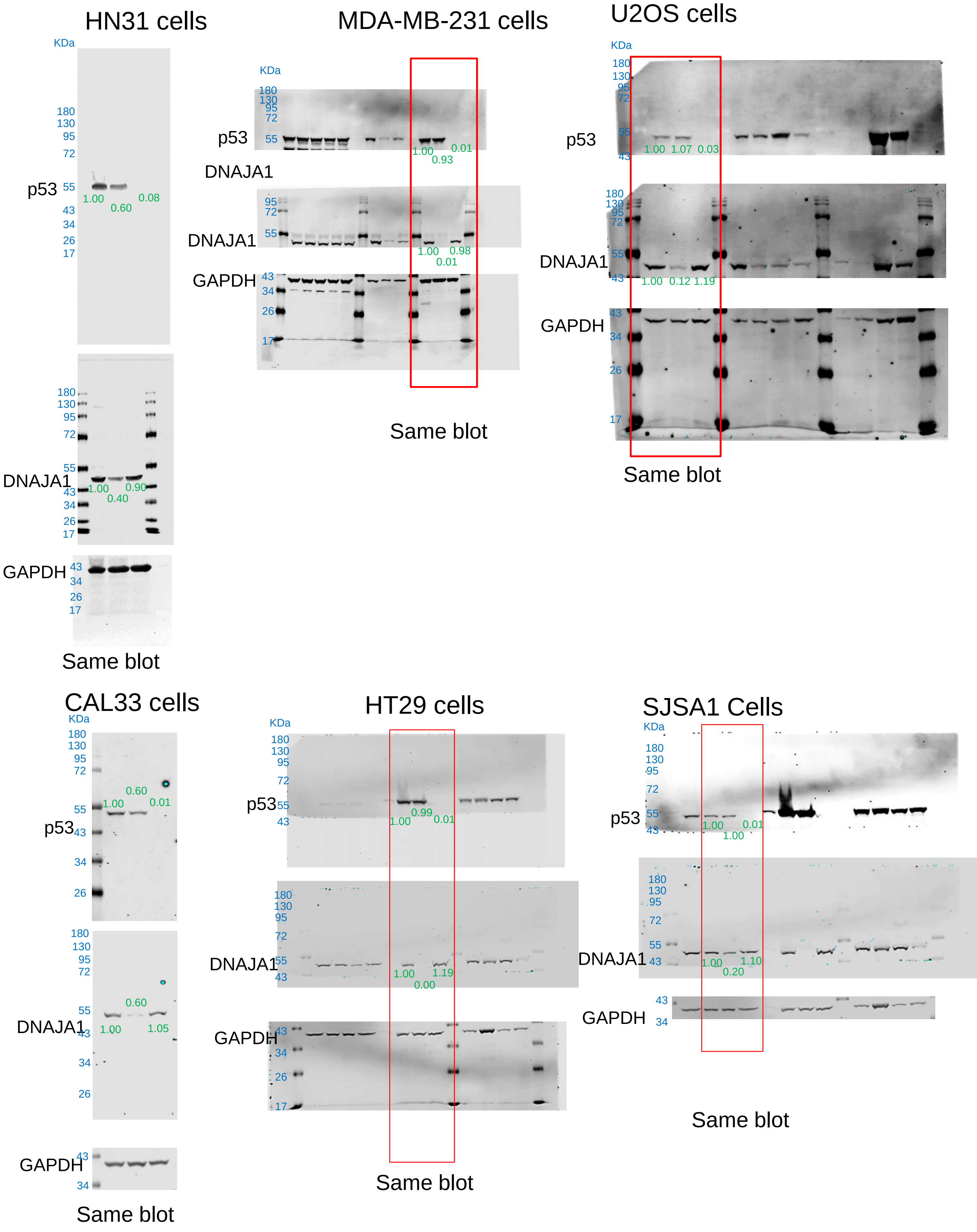

B Original blots of Figure 1A

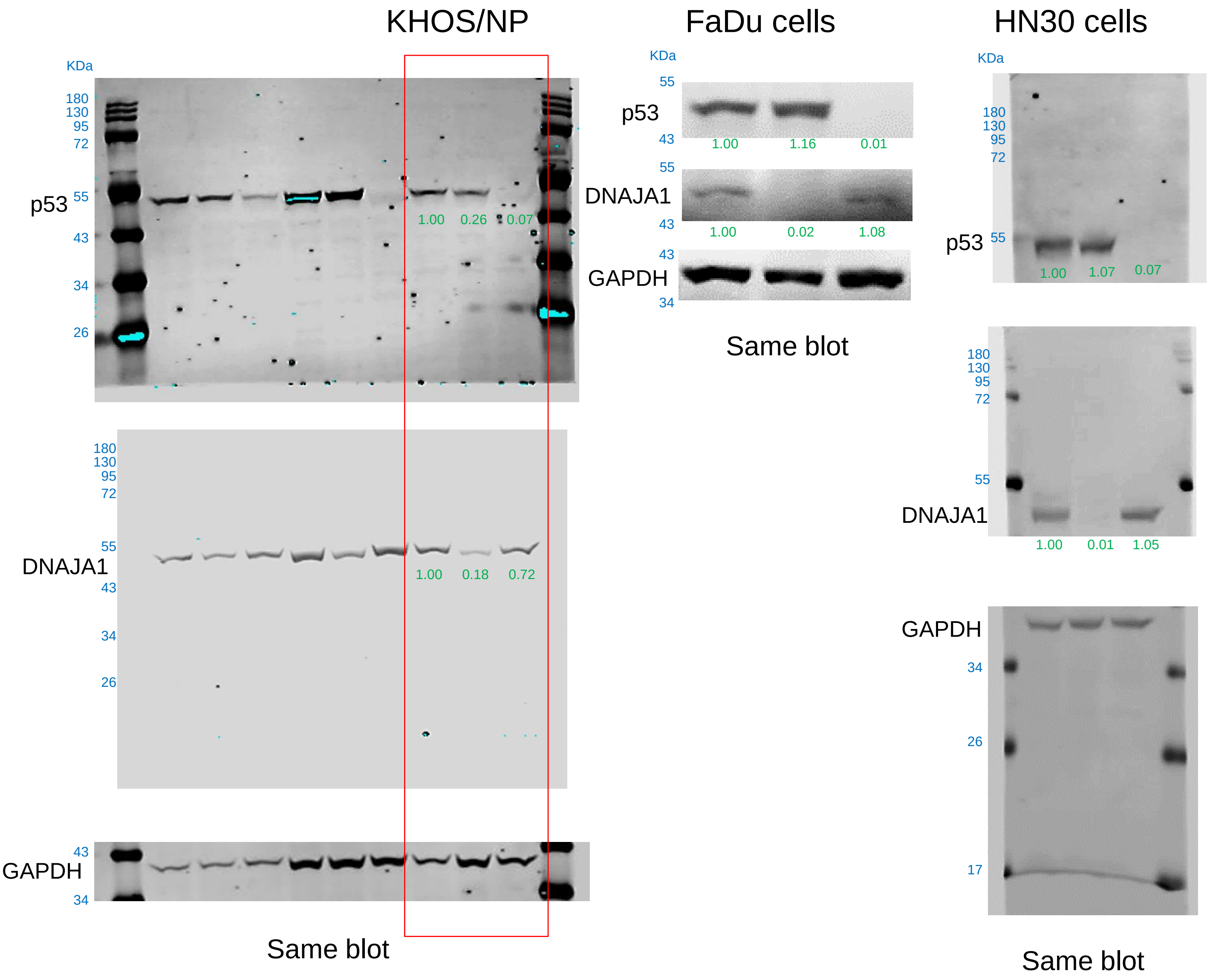

C

PLINH PLIHZ

Original blots of Figure 2B

Cal33 cells

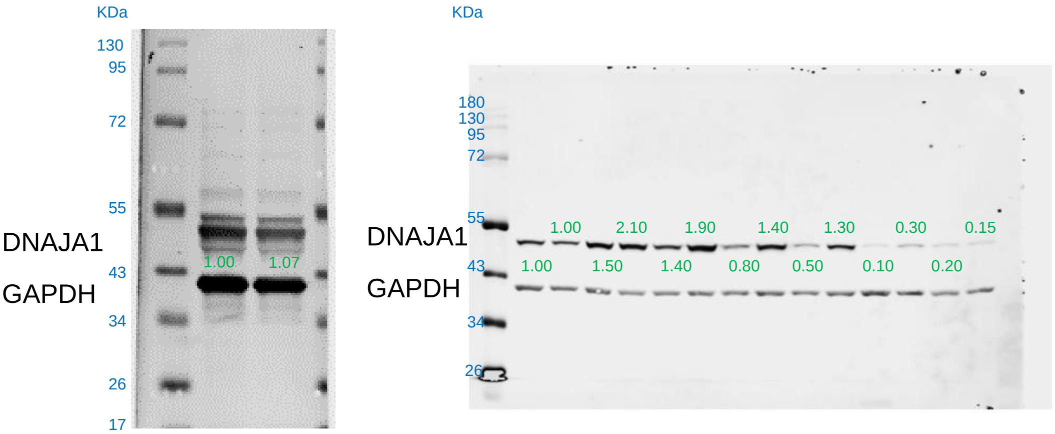

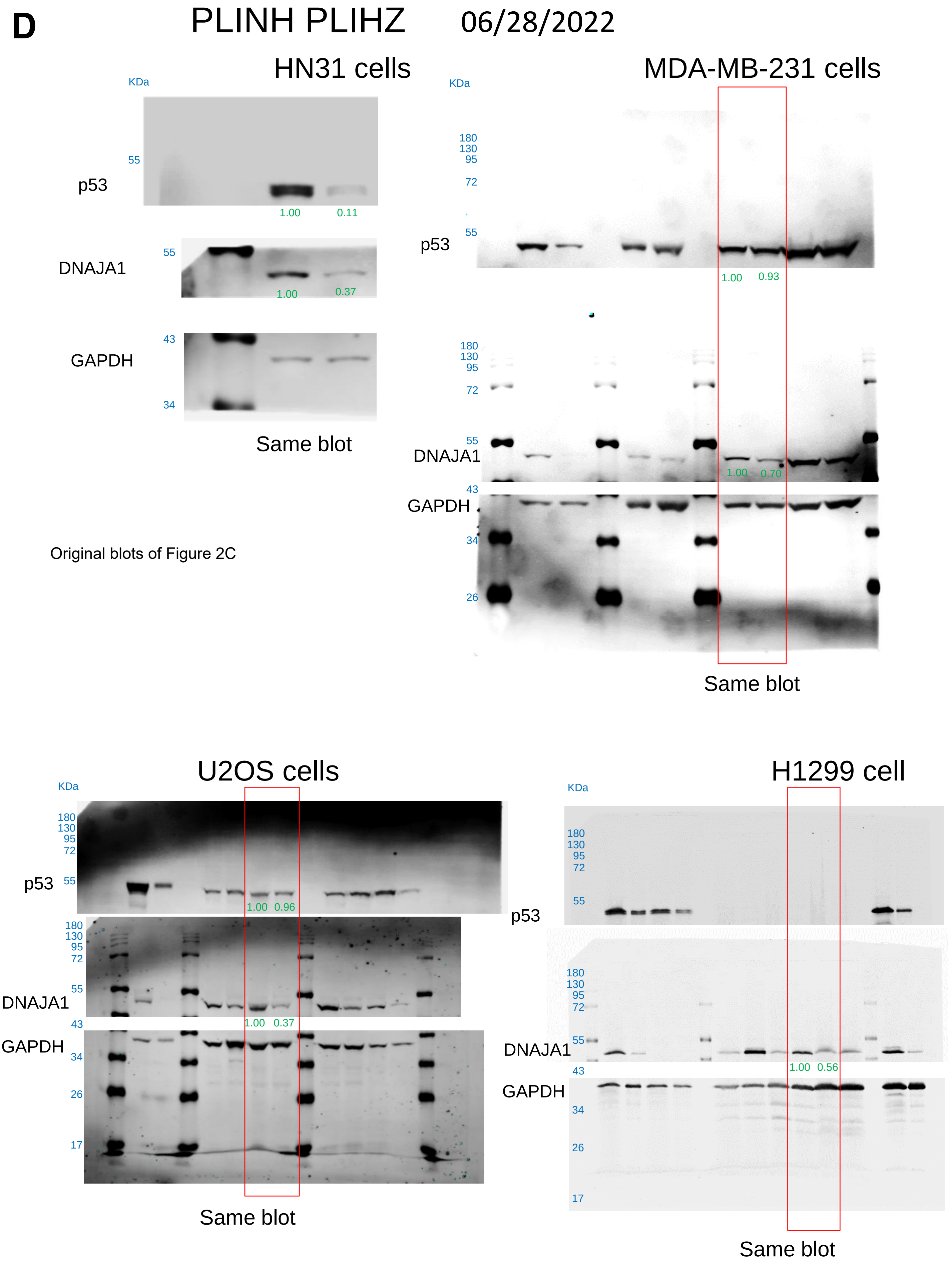
D
PLINH PLIHZ
06/28/2022
HN31 cells
KDa
55
p53
1.00
0.11
DNAJA1
55
1.00
0.37
GAPDH
43
34
Same blot
Original blots of Figure 2C
MDA-MB-231 cells
KDa
180
130
95
72
55
p53
1.00
0.93
180
130
95
72
55
DNAJA1
1.00
0.70
43
GAPDH
34
26
Same blot
U2OS cells
KDa
180
130
95
72
p53
55
1.00
0.96
180
130
95
72
55
DNAJA1
43
1.00
0.37
GAPDH
34
26
17
Same blot
H1299 cell
KDa
180
130
95
72
55
p53
180
130
95
72
55
DNAJA1
43
1.00
0.56
GAPDH
34
26
17
Same blot

E PLTFBH

Original blots of Figure 3B

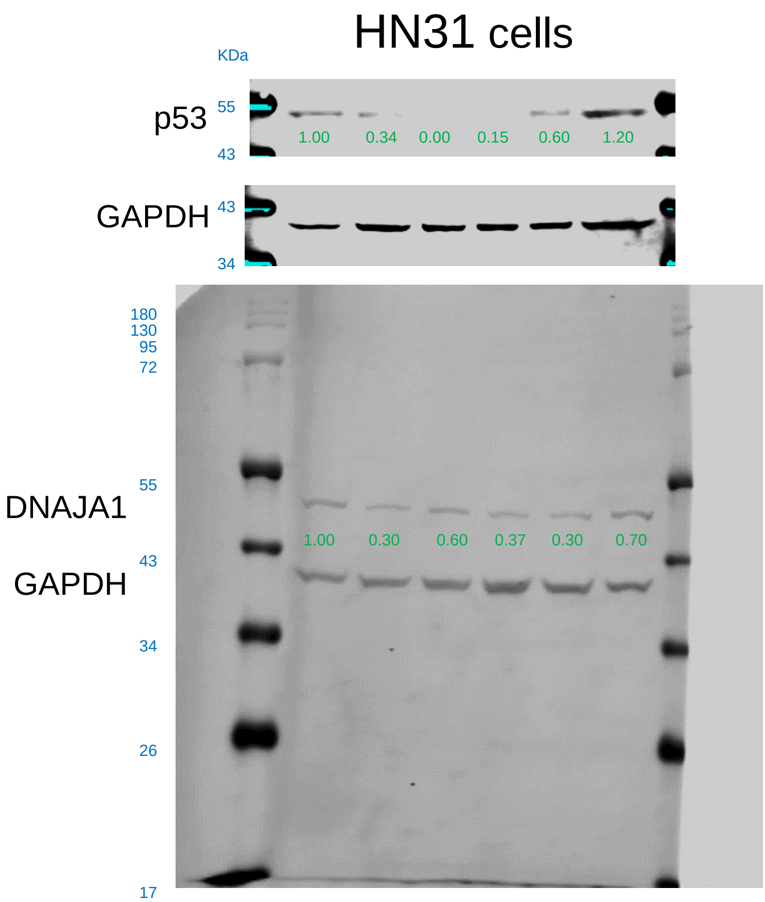

Same blot

F PLTFBH

Original blots of Figure 3C

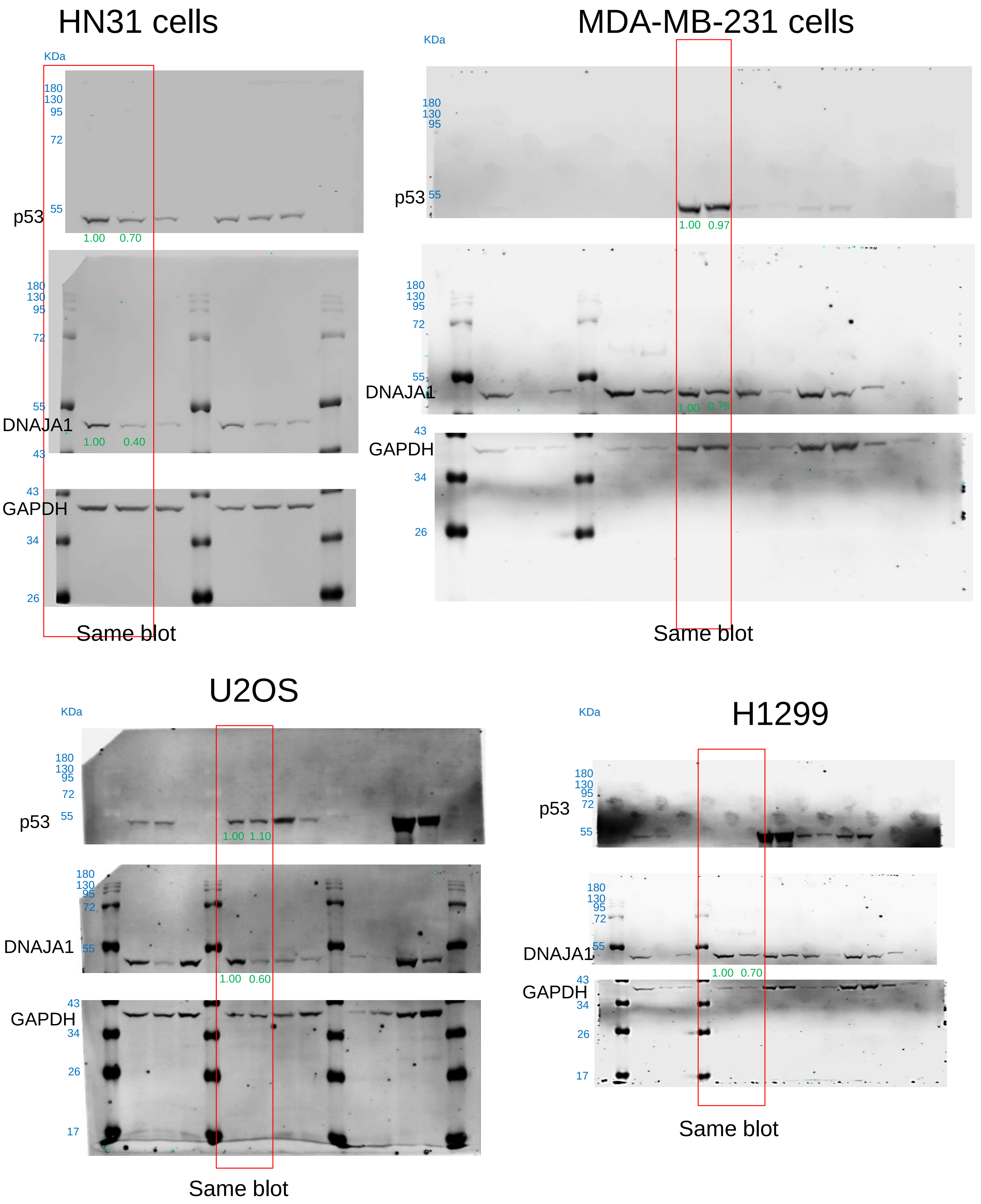

G

PLTFBH

Original blots of Figure 3E

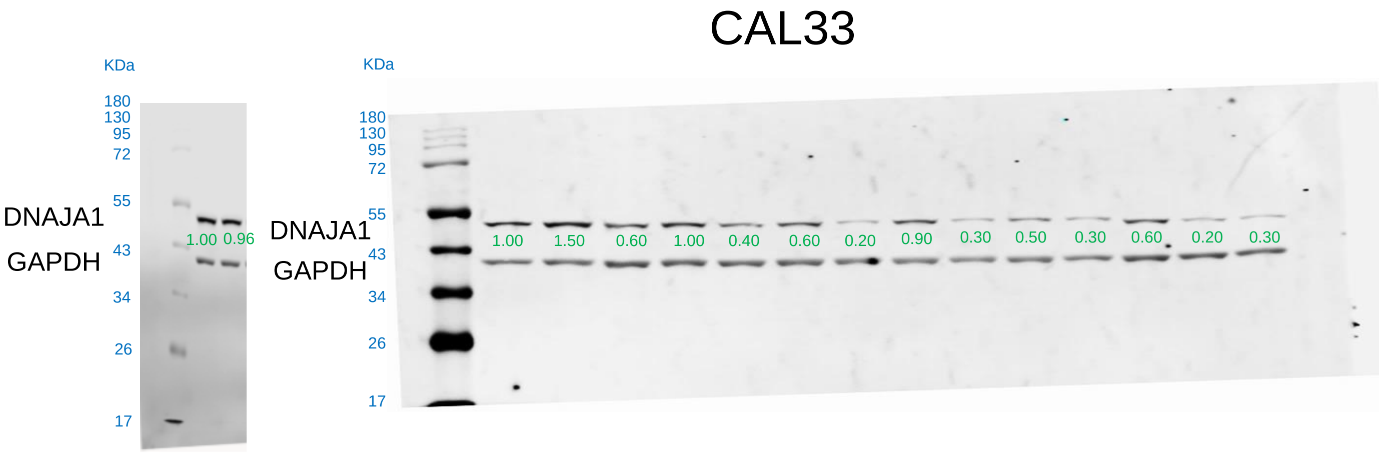

H

Original blots of Figure 3F

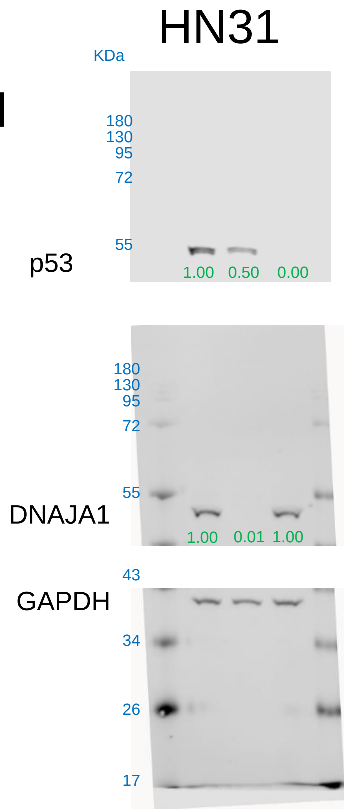

Same blot

Original blots of Figure 4D

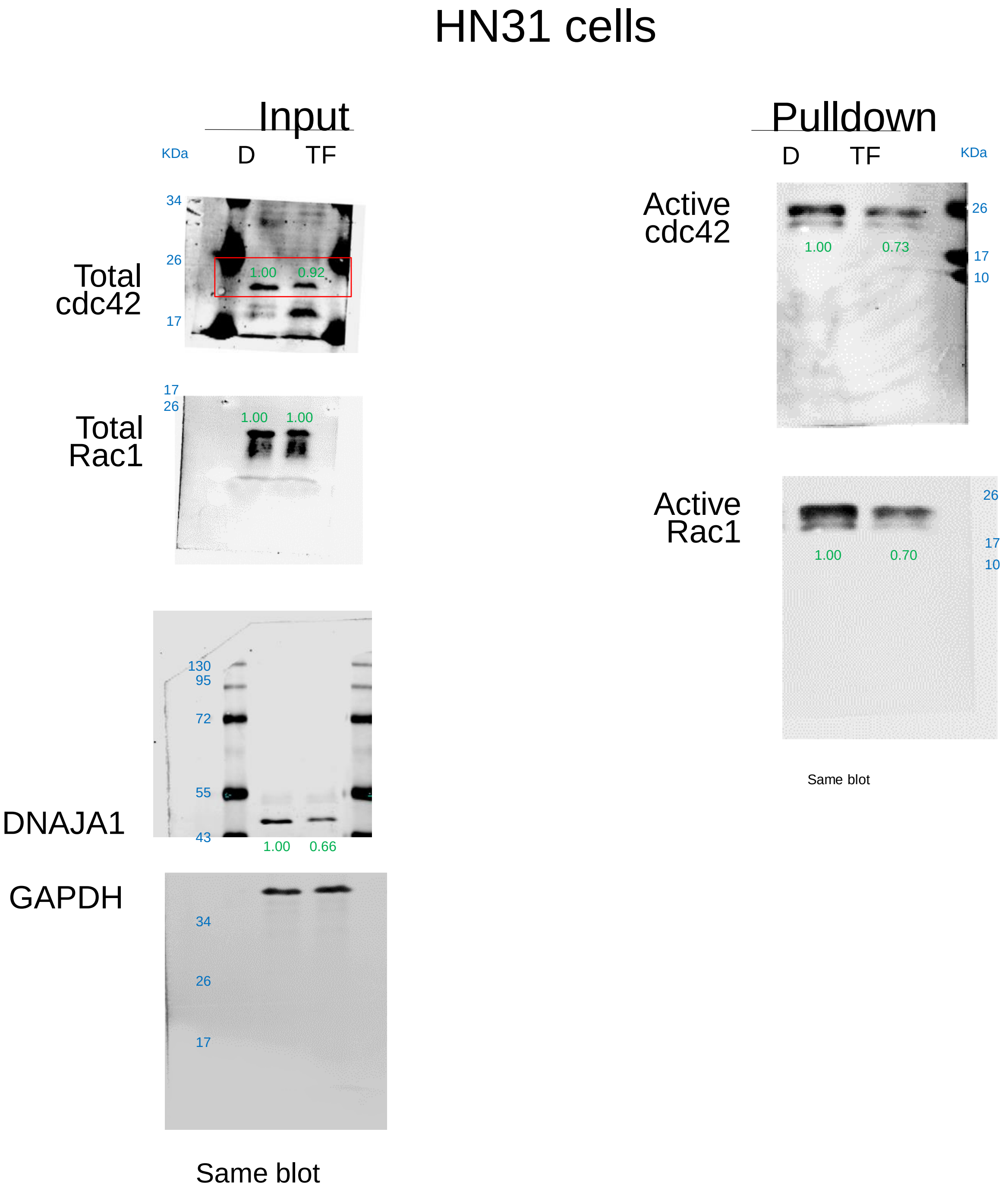

J

Original blots of Figure 5A

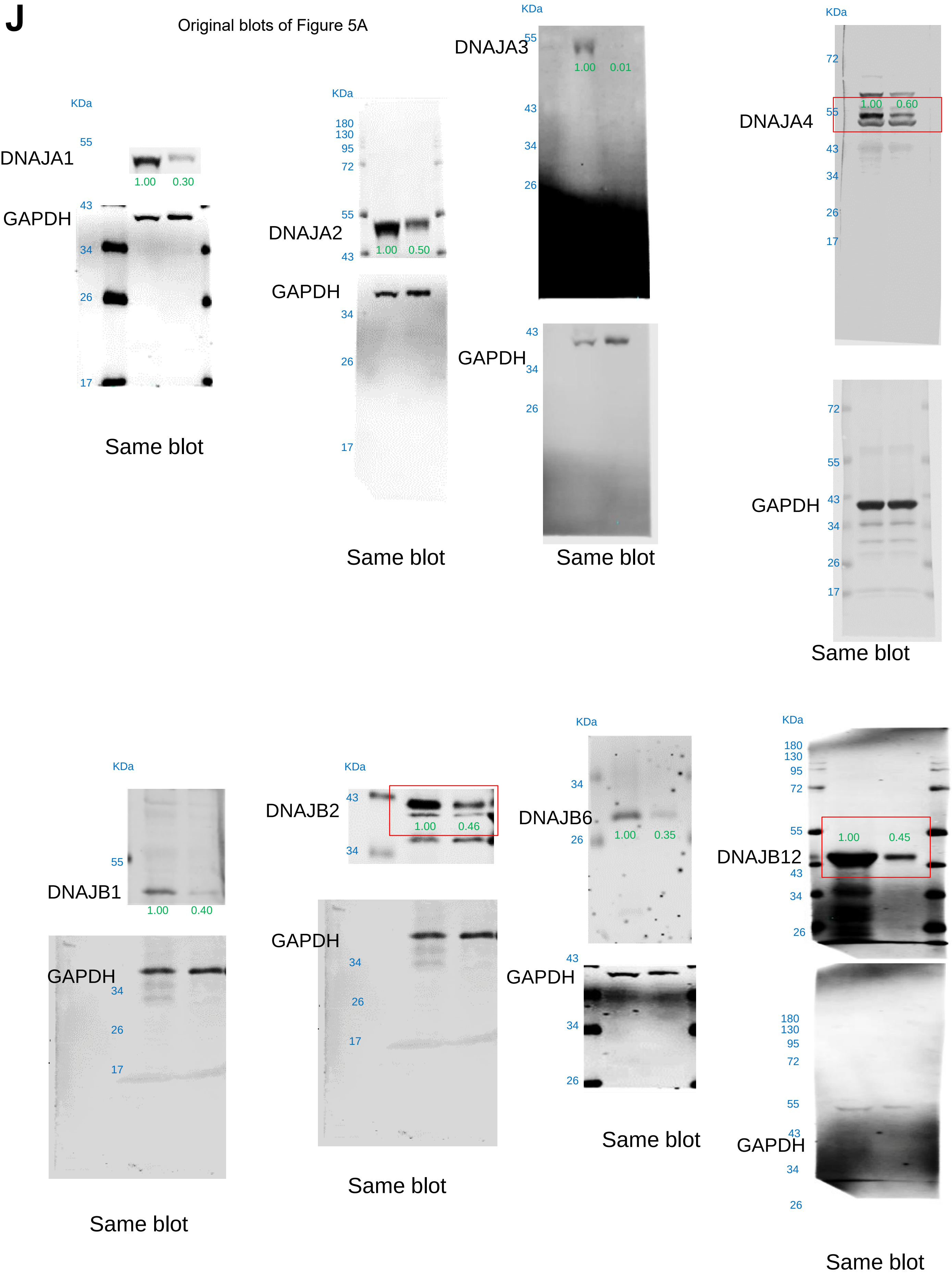

K Original blots of Figure 5A

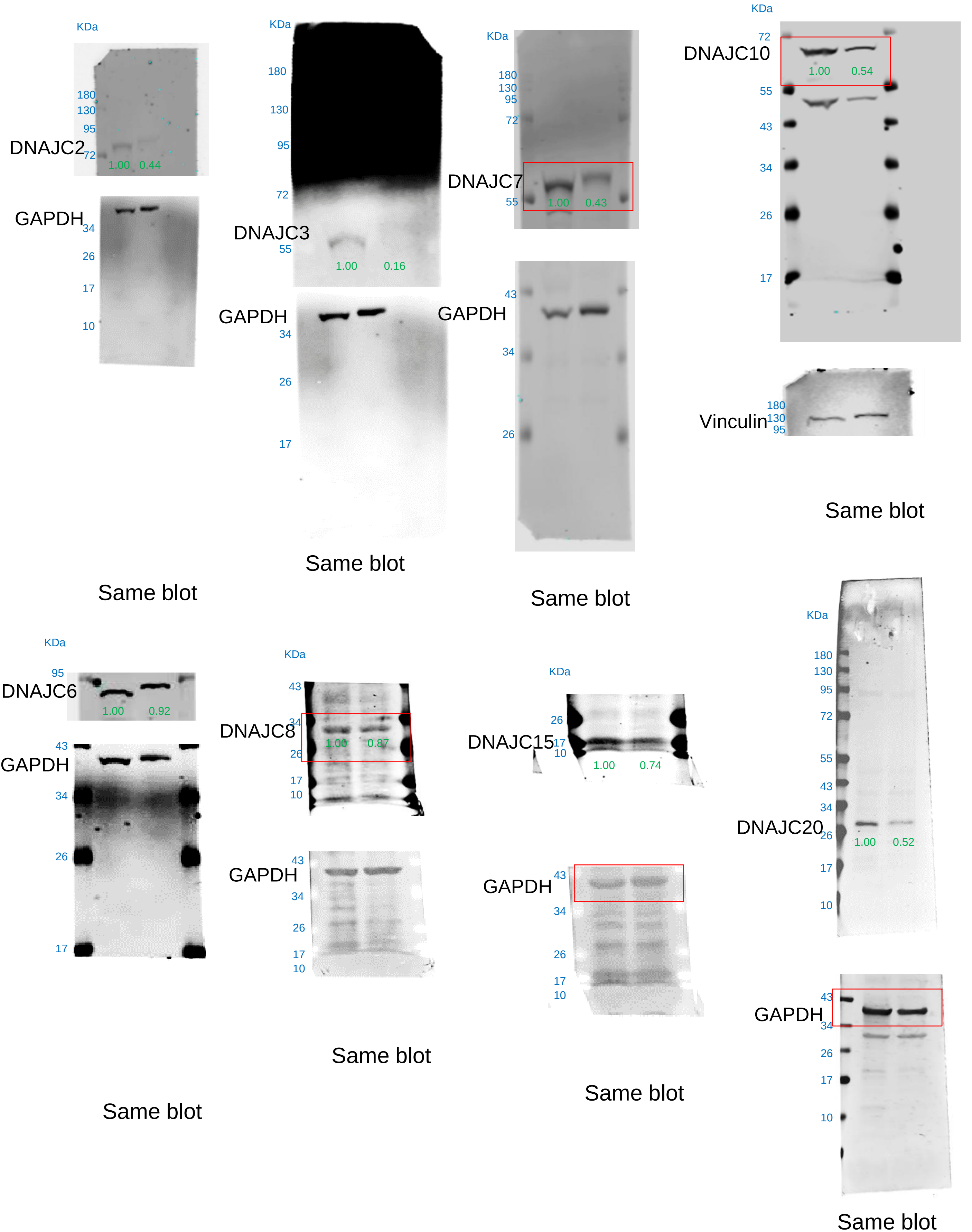

L

Original blots of Figure 5D

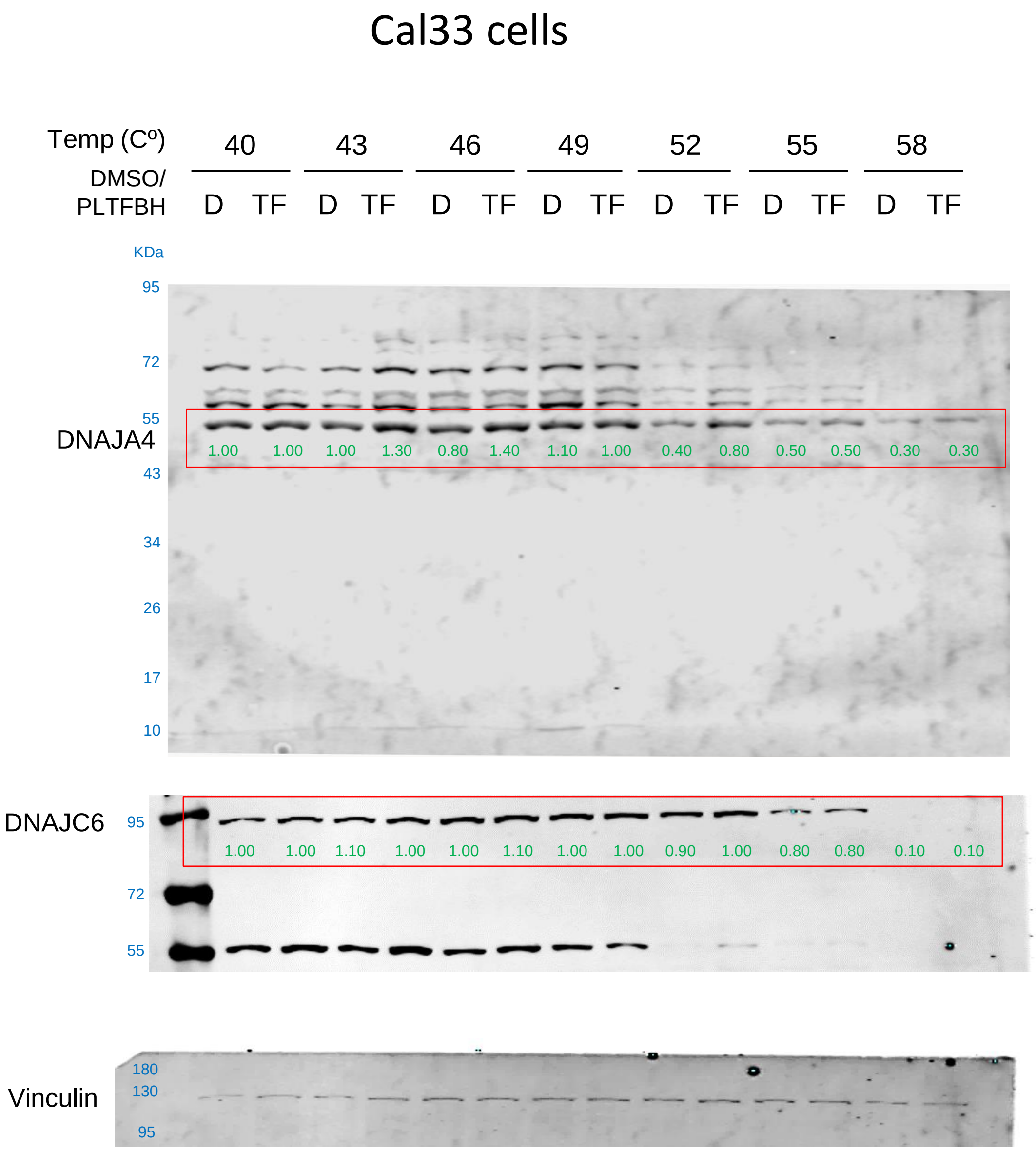

O

HN31 cells

Original blots of Figure 6A

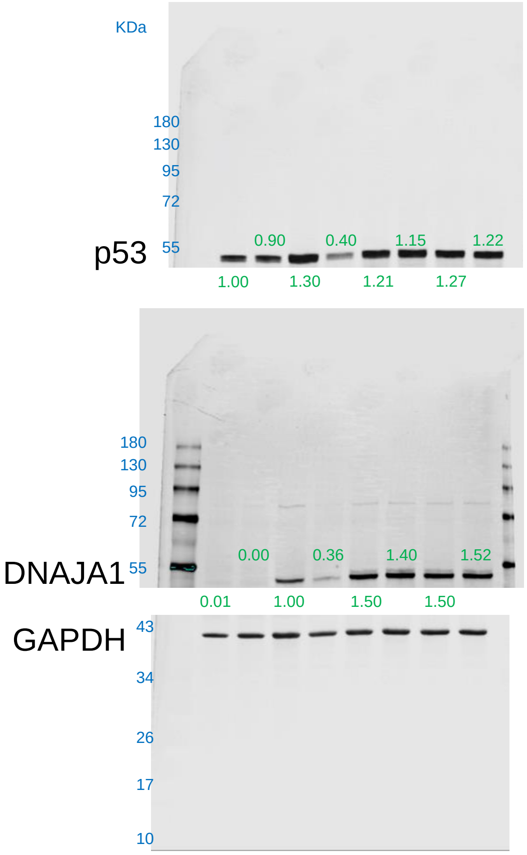

Same blot

P

PLINH PLIHZ

Original blots of Supplementary Figure S2A

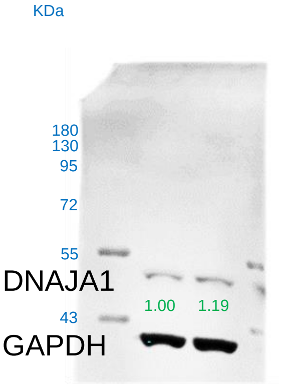

HN31 cells

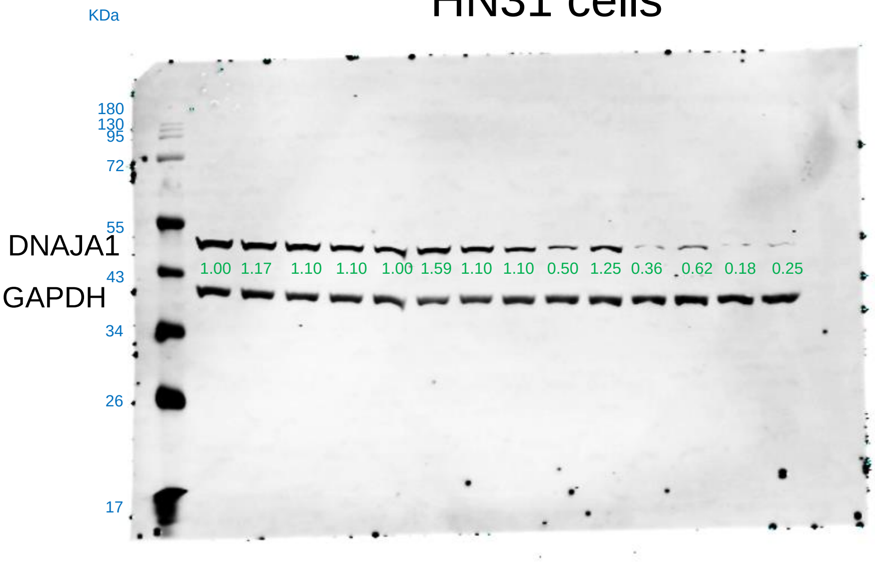

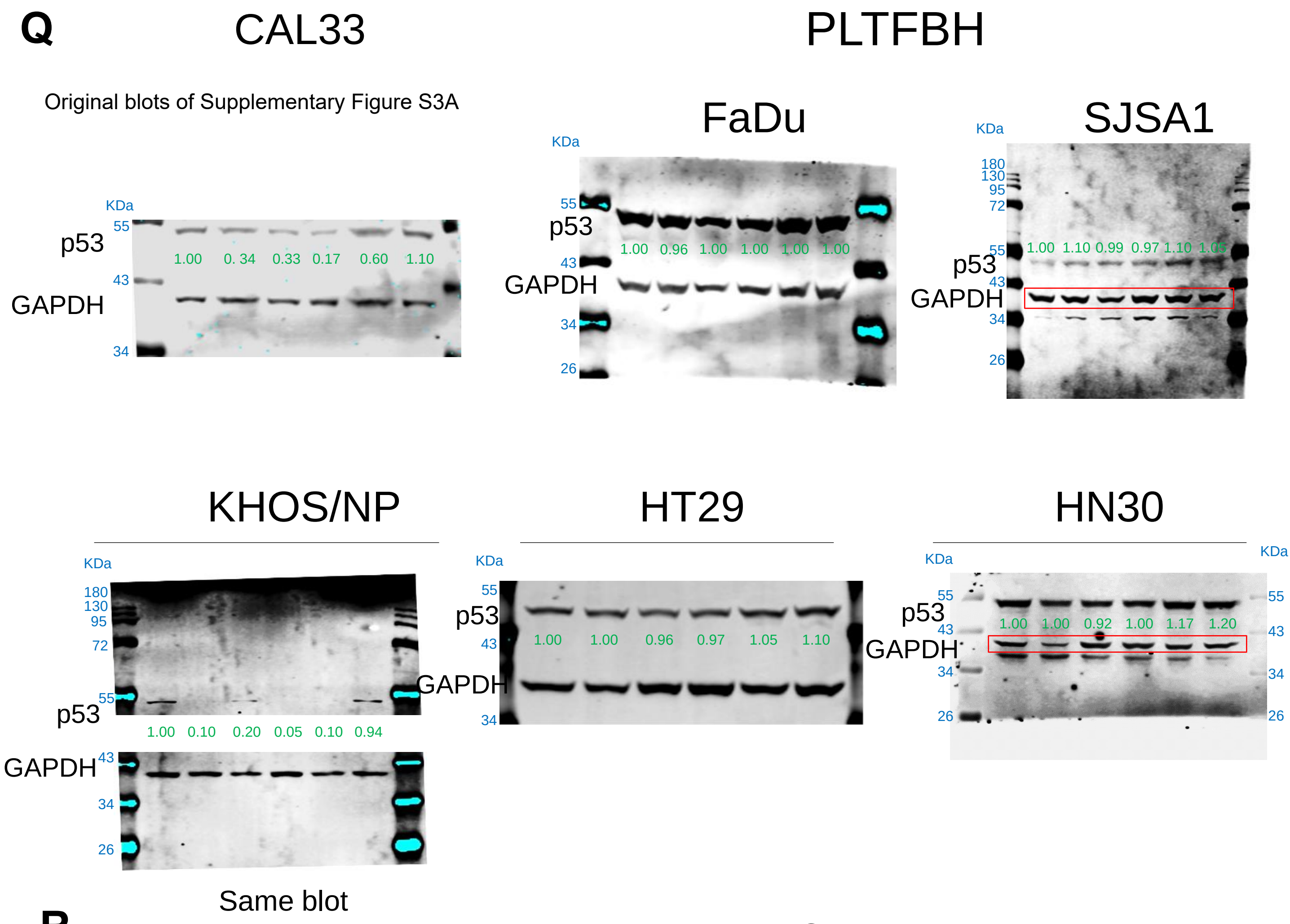

Original blots of Supplementary Figure S3A

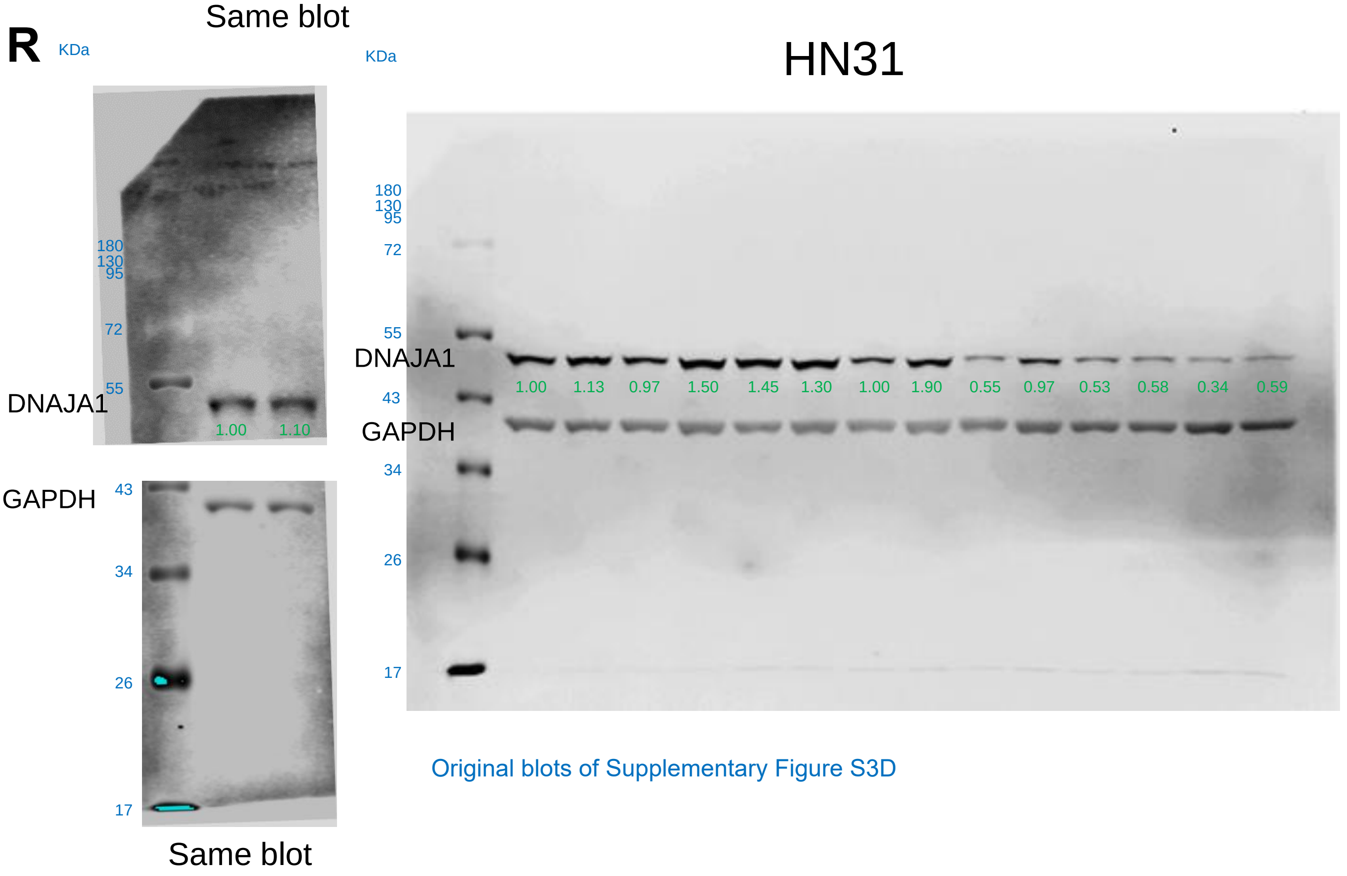

Original blots of Supplementary Figure S3D

S

Original blots of Supplementary Figure S4D

MDA-MB-231

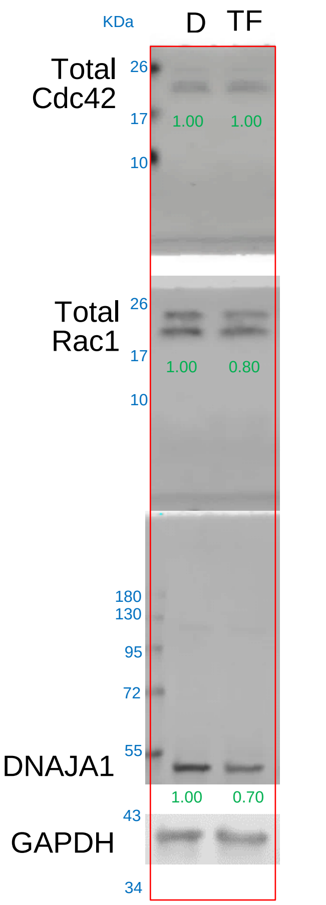

Same blot

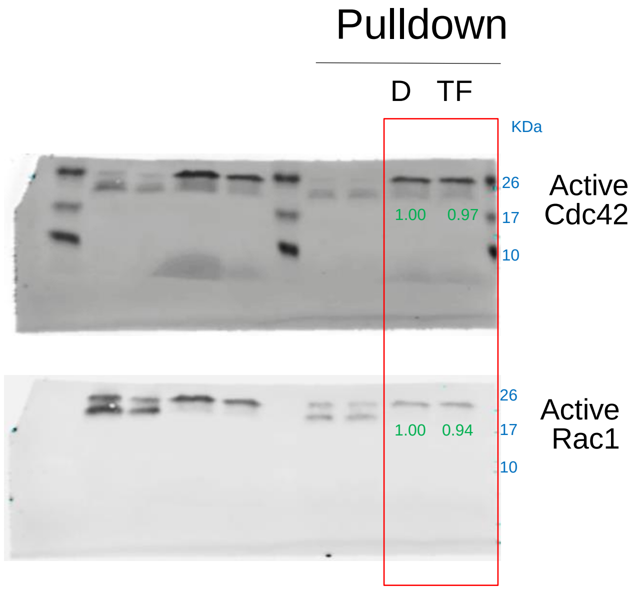

Same blot

T

Original blots of Supplementary Figure S5B

HN31 cells

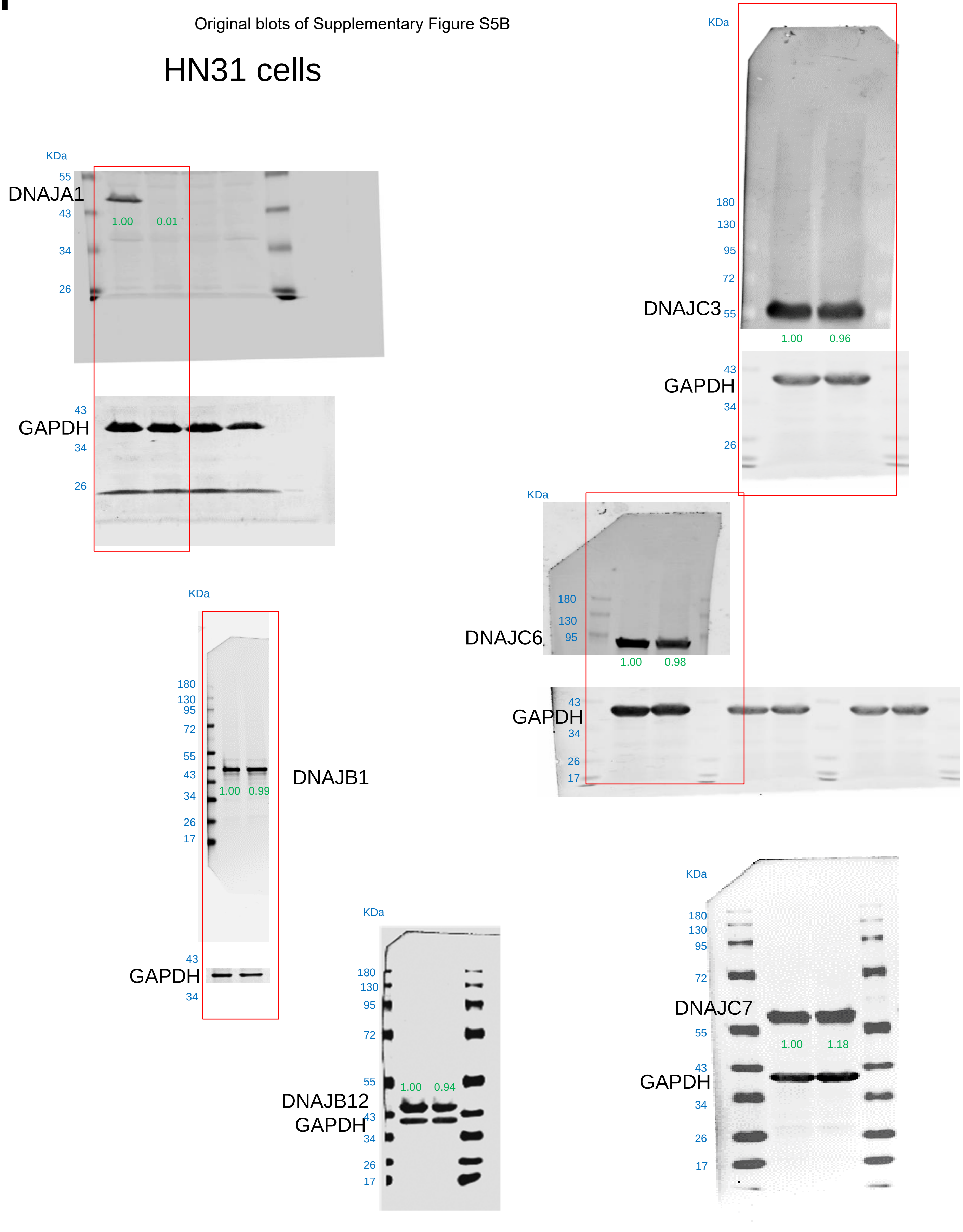

17

U

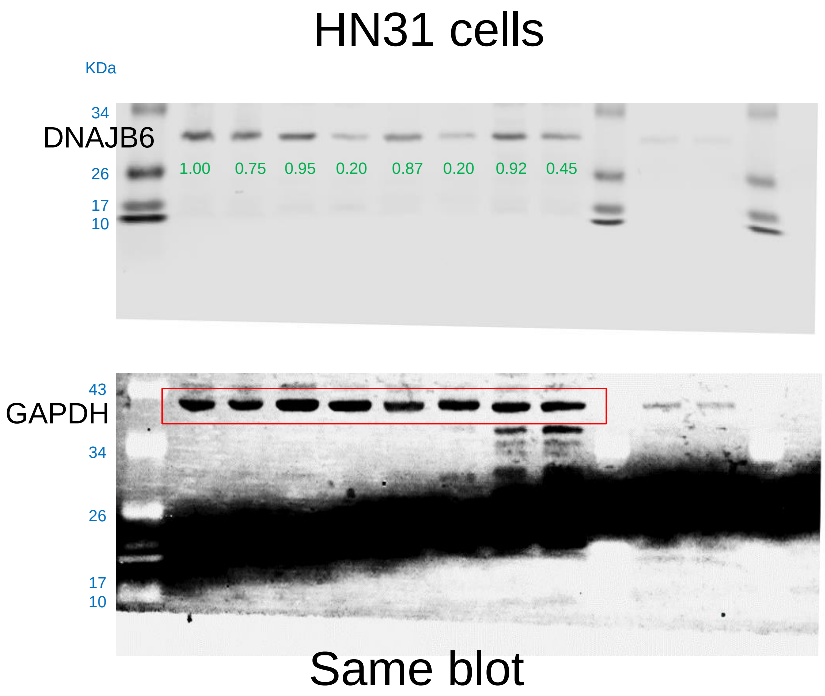

Original blots of Supplementary Figure S6A
