# Supplementary material for: Mutant p53 Depletion by Novel Inhibitors for HSP40/J-Domain Proteins Derived from the Natural Compound Plumbagin
Source: Cancers (Basel). 2022 Aug 29;14(17):4187. doi: 10.3390/cancers14174187 (PMC9454493; doi:10.3390/cancers14174187)
Supplement: Supplementary file 1 [file cancers-14-04187-s001.zip › cancers-1822168-Supplementary Figures.pdf]

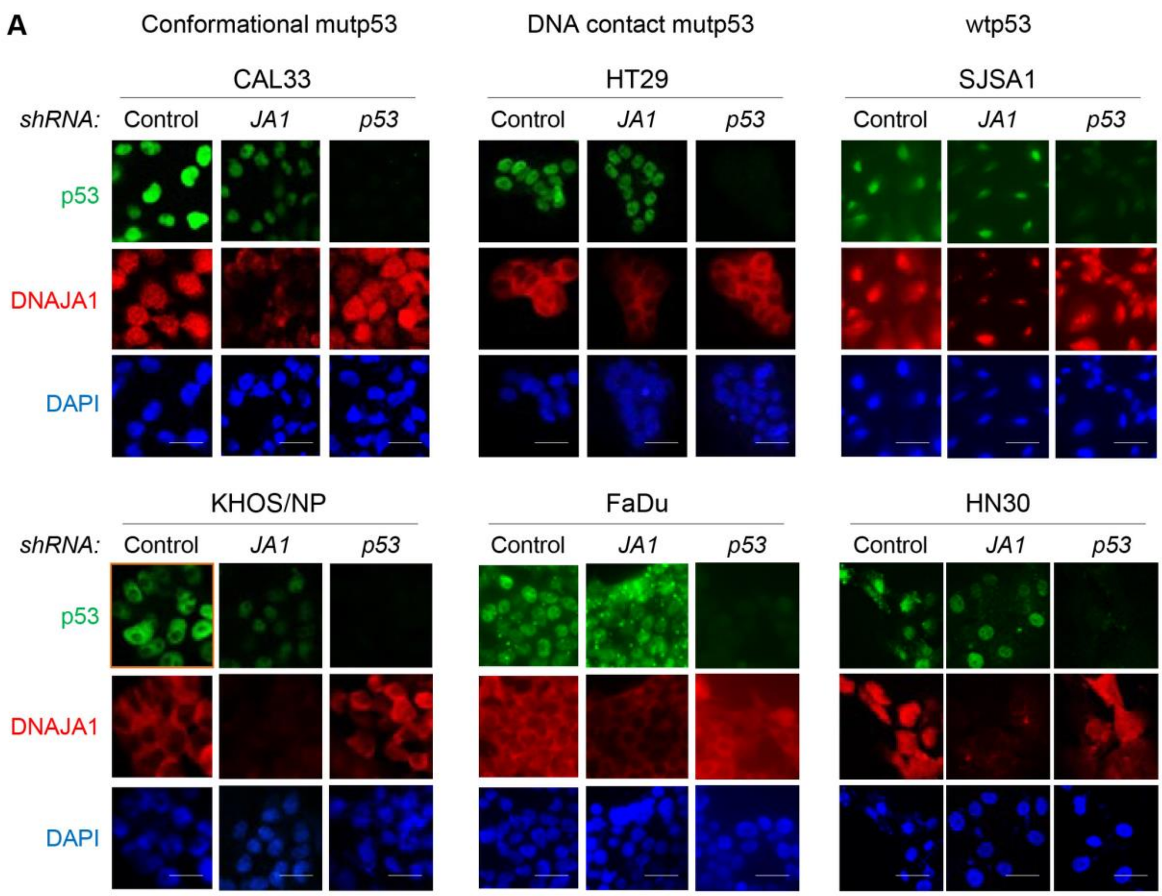

Supplementary Figure S1. Knockdown of DNAJA1 specifically reduces protein levels of conformational mutp53, but not DNA contact mutp53 and wtp53. Immunofluorescence for p53, DNAJA1, and DAPI using multiple cancer cells with different p53 status. Scale bar: 50 μm.

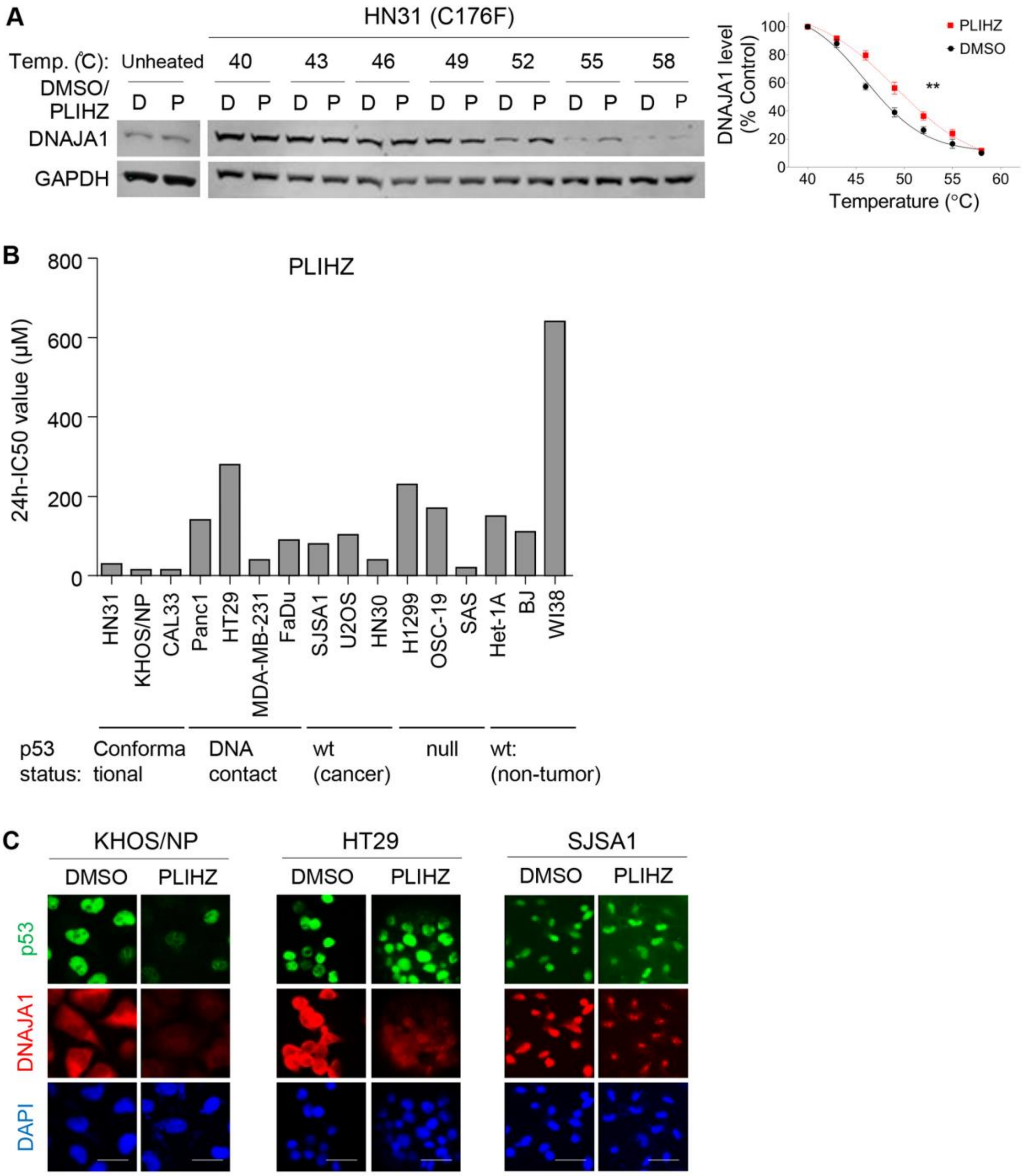

Supplementary Figure S2. Binding of PLIHZ to DNAJA1 and the effects on cell viability and protein levels of DNAJA1 and p53 in multiple human cell lines with different p53 status. (**A**) CETSA showing intracellular binding of PLIHZ to DNAJA1. A representative Western blotting for DNAJA1 and GAPDH using protein extracts from HN31 ($p53^{C176F}$) cells with treatment with DMSO and PLIHZ at 80 μM for 4 h, followed by incubation at different temperatures for 3 minutes (left). A representative blot using protein extracts from unheated cells are also shown. A summarized graph showing normalized DNAJA1 band densities at different temperatures of 40, 43, 46, 49, 52, 55, and 58 °C (right). Mean ± SEM from three independent experiments (n=3). ** $p < 0.01$ for two-way ANOVA. (**B**) Summary of 24h-IC50 values of PLIHZ, determined by MTT assays, in multiple human cell lines with different p53 status as indicated. (**C**) Immunofluorescence for p53, DNAJA1, and DAPI, using indicated cells treated with DMSO or PLIHZ at ~1/2 of 24h-IC50 for 24 h. Scale bar: 50 μm.

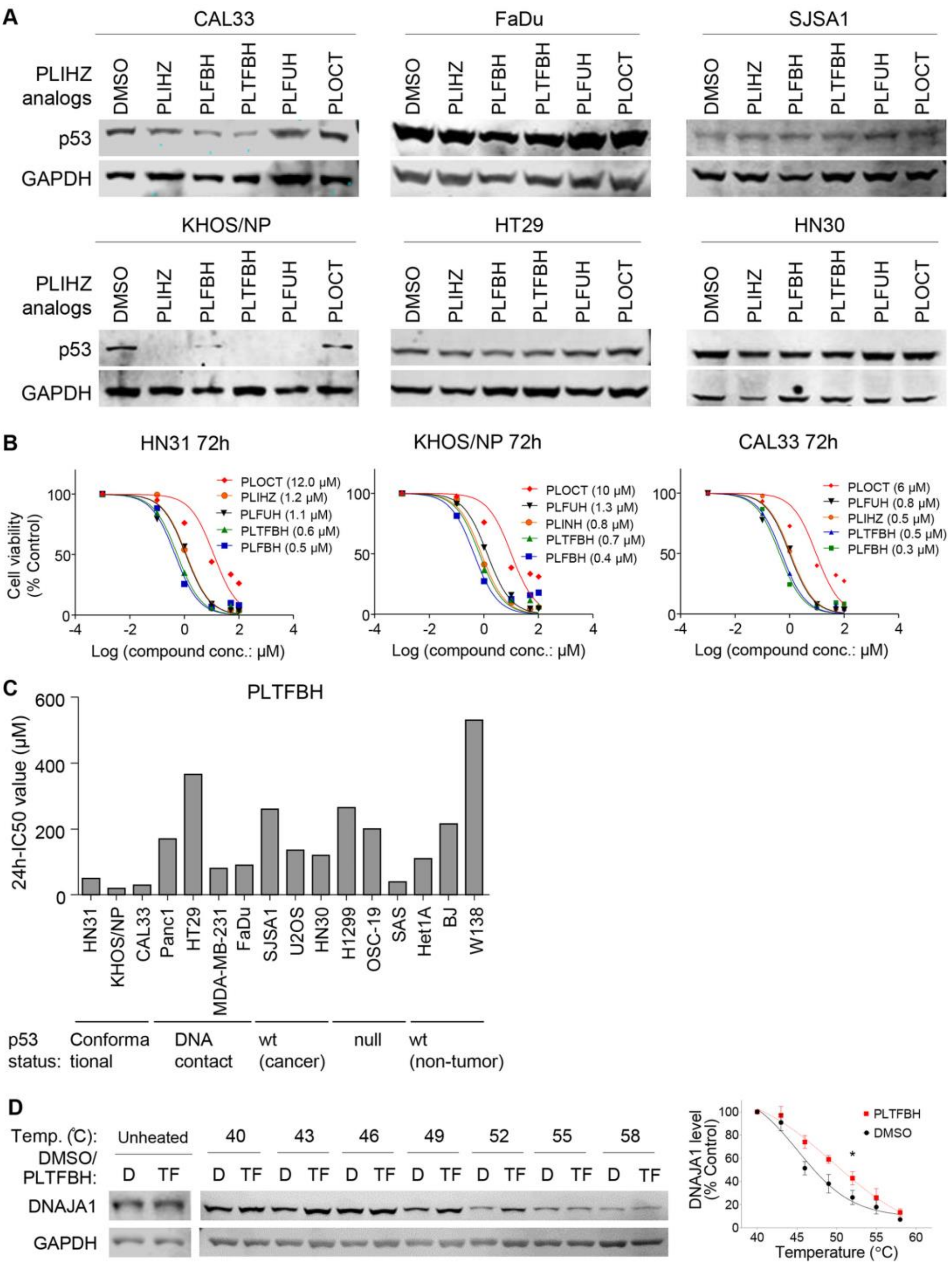

Supplementary Figure S3. Effects of PLIHZ analogs on mutp53 levels, cell viability, and binding to DNAJA1. (**A**) Western blotting for p53 and GAPDH using indicated cell lines with different p53 status following treatment with different PLIHZ analogs at 40 μM for 24 h. (**B**) Summary of MTT assays (72 h) using HN31, KHOS/NP and CAL33 cells treated with different concentrations of PLIHZ analogs for 72 h. Mean ± SEM from three independent experiments (n=3). The IC50 value of each compound is shown on the right. (**C**) Summary of 24h-IC50 values of PLTFBH, determined by MTT assays, in multiple human cancer and non-tumor cell lines with different p53 status as indicated. (**D**) CETSA showing intracellular binding of PLTFBH to DNAJA1. A representative Western blotting for DNAJA1 and GAPDH using protein extracts from HN31 ($p53^{C176F}$) cells with treatment with DMSO and PLTFBH at 80 μM for 4 h, followed by incubation at different temperatures for 3 minutes (left). A summarized graph (right). Mean ± SEM from three independent experiments (n=3). * $p < 0.05$ for two-way ANOVA.

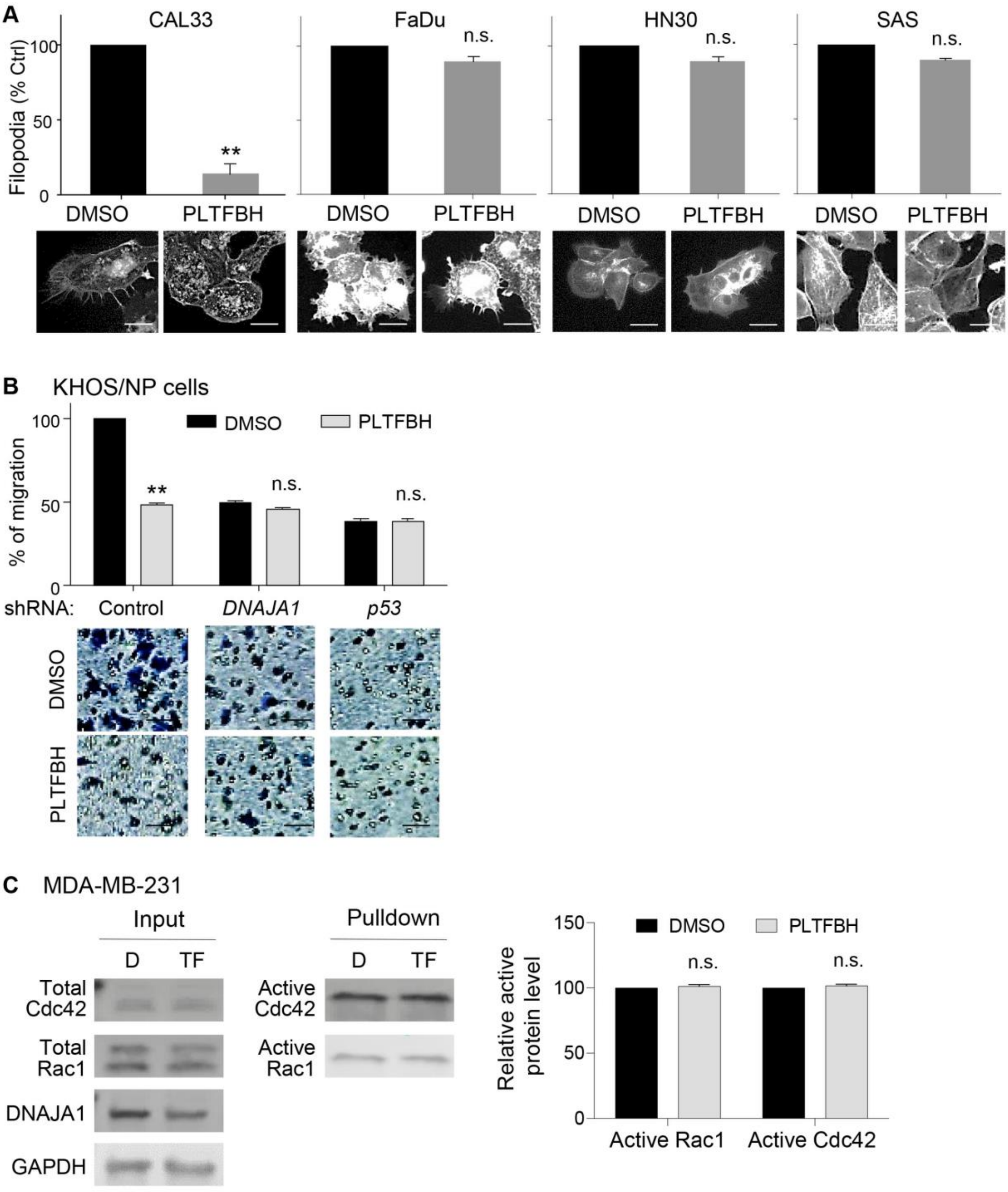

Supplementary Figure S4. PLTFBH inhibits migratory potential of cancer cells in a manner dependent on DNAJA1 and conformational mutp53. (**A**) F-actin staining showing inhibition of filopodia formation in CAL33 cells, but not FaDu, HN30, and SAS cells, by PLTFBH. Top: summarized graph. Bottom: representative images. Mean ± SEM from three independent experiments (n=3). ** $p < 0.01$ for two-tailed Student's *t*-test. n.s.: not significant. Scale bar: 10 μm. (**B**) Transwell migration assays using *DNAJA1*- or *p53*-knockdown KHOS/NP cells treated with PLTFBH at ~1/2 IC50 for 12 h. Cells were pre-treated with PLTFBH for 12 h. Mean ± SEM from three independent experiments (n=3). ** $p < 0.01$ for two-tailed Student's *t*-test. n.s.: not significant. Scale bar: 100 μm. (**C**) Rac1/Cdc42 activation assays following pulldown of active Rac1 and Cdc42 using protein extracts from MDA-MB-231 cells treated with DMSO (D) or PLTFBH (TF) at ~1/2 of 24h-IC50. Left: representative immunoblots. Right: summarized graph. n.s.: not significant for two-tailed Student's *t*-test.

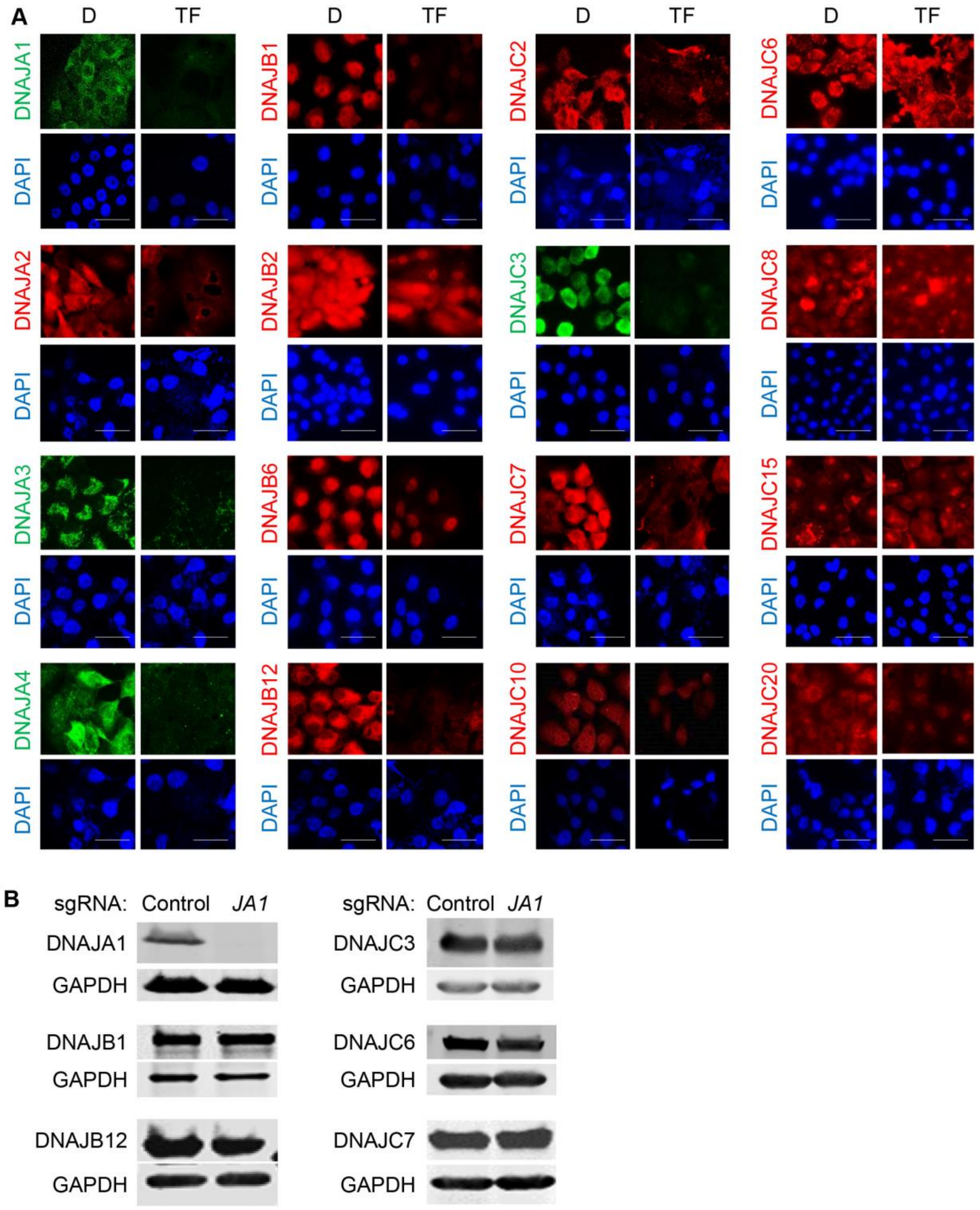

Supplementary Figure S5. PLTFBH selectively decreases protein levels of certain members of HSP40/JDPs. (**A**) Immunofluorescence for several members of HSP40/JDPs and GAPDH using HN31 cells treated with DMSO (D) or PLTFBH (TF) at ~1/2 IC50 for 24 h. Scale bar: 50 μm. (**B**) Western blotting for DNAJA1, DNAJB1, DNAJB12, DNAJC3, DNAJC6, DNAJC7, and GAPDH using HN31 cells with or without *DNAJA1* knockout (*JA1*).

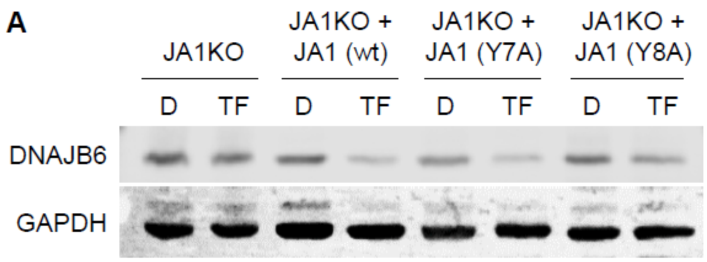

Supplementary Figure S6. Mutations at Y7 and Y8 residues in DNAJA1 abrogates the ability of PLTFBH to deplete DNAJA1 and conformational mutp53. (**A**) Western blotting to detect endogenous DNAJB6 and GAPDH using DNAJA1-KO HN31 cells (JA1KO) expressing exogenous wild-type (wt), Y7A mutant (Y7A), and Y8A mutant (Y8A) DNAJA1, treated with DMSO (D) or PLTFBH (TF) at ~1/2 IC50 for 24 h.
